# The Processing Mechanism of Repetitive Practice Affecting Time-Based Prospective Memory

**DOI:** 10.3390/bs13090750

**Published:** 2023-09-10

**Authors:** Jiaqun Gan, Yunfei Guo, Enguo Wang

**Affiliations:** Institute of Psychology and Behavior, Henan University, Kaifeng 475000, China; ganjiaqun@henu.edu.cn (J.G.); gyfhenu@126.com (Y.G.)

**Keywords:** time-based prospective memory, behavior training, attention consumption, internal attention, external attention

## Abstract

Time-based prospective memory (TBPM) refers to performing intended actions at a specific time in the future. The TBPM task is very common in daily life, and whether it can be successfully completed can affect our quality of life. Repeated behavior training can usually improve social cognitive performance, and this study focused on exploring whether TBPM performance could improve with repeated behavior training. Meanwhile, we also focused on whether behavior training could reduce attention consumption, both internal and external, attention on TBPM tasks. In this study, a single-factor between-subject design was adopted. Seventy-three undergraduates were assigned to three groups: the control group, the baseline group, and the experimental group. The baseline group only needs to perform ongoing tasks, so the ongoing task performance of the control group will not be affected by TBPM tasks. The control group needs to perform both ongoing and TBPM tasks without practice. The experimental group needs to perform both ongoing and TBPM tasks after 30 exercises. The ongoing task is a typical working memory task. The TBPM task was to press a “1” button every 1 min. The results showed that the performance of ongoing tasks in the baseline group, experimental group, and control group decreased sequentially, and the experimental group had less time monitoring than the control group. The results indicated that behavior training could reduce attention consumption in both internal attention and external attention, but it could not reach the level of automatic processing.

## 1. Introduction

Prospective memory is the ability to perform something that has been planned for future situations [1]. Event-based prospective memory (EBPM) and time-based prospective memory (TBPM) are two types of prospective memory artificially divided according to different types of cues. EBPM needs to be performed when there is a clear cue from the outside world (e.g., you should remember to buy some vegetables when passing the market). TBPM needs to be performed at a specific time (e.g., you should remember to have a meeting at 3:30 tomorrow afternoon). Prospective memory tasks are very common in our lives. The evidence suggests that more than half of our daily memory failures are prospective memory failures [2]. In view of the importance of prospective memory, how to improve prospective memory performance should be paid more attention. Research has shown that behavior training is an effective method for improving prospective memory performance [3]. Borm and Kliegel divided behavior training into strategy training and cognitive process training [3]. Strategy training mainly trains individuals to use efficient strategies. Strategy training is usually an auxiliary method that promotes TBPM performance through external means. The effectiveness of this method is short-lived and targeted. Cognitive processing training focuses on how to improve individual cognitive ability. This approach typically improves TBPM performance by enhancing individual abilities. For example, TBPM tasks heavily rely on individuals’ working memory ability, inhibition ability, and attention control ability. If the performance of TBPM is improved by training the above abilities, then it belongs to cognitive processing training. Repetitive practice does not focus on improving individual abilities but directly enhances TBPM abilities themselves. This is also typical cognitive processing training. This study mainly focused on the role of repeated behavior training in improving TBPM performance.

A large number of studies have found that repeated behavior training can improve individual EBPM performance. Some studies used a Virtual Week program (simulating a week’s activities by rolling dice and moving the grids) to explore the effect of task regularity on prospective memory. The results showed that the performance of the EBPM task that occurred multiple times was significantly better than that of the EBPM task that occurred only once [4,5]. Guo et al. focused on the impact of different encoding methods on EBPM and found that training for four trials can significantly improve the prospective memory performance of college students [6]. Meanwhile, the training effect was not only the best, but it also did not interfere with ongoing tasks. They suggested that practice training reduced individual attention consumption on EBPM tasks and promoted automatic task processing. Another study used the event-related potentials (ERPs) technique to investigate whether brain activity related to EBPM in the elderly showed stable and lasting plasticity after practice. The results showed that the overall activation of ERP components (N300, P3, PP) in the posterior parietal cortex of the elderly was found to decrease, which indicated that EBPM tasks were retrieved in a relatively automatic processing state [7]. Based on the above evidence, behavior training can improve EBPM performance while reducing attention consumption.

Several studies have also found that repeated behavior training can improve TBPM performance in healthy-aging adults [4,5,8]. Two of the studies used the Virtual Week program, a game similar to flying chess in which participants needed to roll dice and move squares, with each circle counting as one day, simulating a total of five days of life. It was found that in the college and elderly groups, the TBPM task that was repeated five times performed better than the TBPM task that only appeared once [4,5]. Guo et al. adopted the working memory task as the ongoing task and found that the TBPM tasks that occurred many times in college students had better behavior performance [8]. Although existing studies have consistently found the promoting effect of repeated behavior training on TBPM, these studies have not delved into the mechanism behind this promoting effect. Compared to EBPM, TBPM does not have clear external cues, and its processing requires more self-initiated attention resources [9]. Repeated TBPM training makes it difficult to achieve automated processing levels. But repetitive execution of TBPM tasks involves multiple extractions of TBPM intentions, and retrieval practice promotes individuals’ spontaneous extraction of memory content and reduces their attention consumption [10,11]. Therefore, multiple behavioral training sessions should improve the automated processing level of TBPM intention, thereby reducing attention consumption during the goal-maintenance period.

TBPM mainly involves two aspect abilities of time information processing and retrospective memory [12]. It is clear that retrospective memory can reduce attention consumption through repeated behavior training [13,14]. However, compared with EBPM, TBPM does not have clear external cues, and the recognition of TBPM cues always requires self-initiated attention resources [15]. Although the recognition of TBPM cues cannot reach the level of spontaneous retrieval, behavior training may reduce attention consumption through an improvement in time estimation ability, thus improving TBPM performance. The time information processing ability of TBPM also includes two aspects. One is external attention, which mainly refers to the individual’s attention to external time information. The other is internal attention, which mainly refers to the efforts made by individuals in the processing of internal time information such as time estimation [16,17]. Laboratory studies of prospective memory usually require individuals to perform both ongoing tasks and prospective memory tasks simultaneously, which makes the two tasks compete for limited cognitive resources simultaneously [18]. Therefore, prospective memory tasks will interfere with ongoing tasks, and the performance of ongoing tasks is usually used to reflect an individual’s internal attention level, while the external attention level is usually reflected by the frequency of time monitoring [17,19,20,21].

The purpose of this study was to explore the processing mechanisms of behavior training that affect TBPM. Specifically, this study focused on whether behavior training can reduce individuals’ attention consumption in terms of internal attention and external attention. We hypothesized that behavior training would reduce both internal and external attention. However, since TBPM always requires self-initiated attention resources, the behavior training of TBPM tasks may not enable prospective memory responses to reach a state of spontaneous retrieval. In this study, the processing mechanism of TBPM with behavior training was firstly explored by paying attention to the changes in internal attention and external attention, which could help us understand the mechanism of TBPM plasticity from the perspective of attention.

## 2. Methods

### 2.1. Participants

Seventy-three undergraduates (aged 18 to 23; *M*_age_ = 19.89, *SD* = 1.41) participated in this experiment. They were randomly assigned to the baseline group (25 participants; 13 women), the control group (25 participants; 15 women), and the experimental group (23 participants; 13 women). All participants had normal vision or corrected vision, no color blindness, and were right-handed. The general content and specific process of the experiment were explained to the participants before they started the experiment. This study was reviewed and approved by Institutional Review Board of Henan Provincial Key Laboratory of Psychology and Behavior.

### 2.2. Tasks

**Figure 1:**
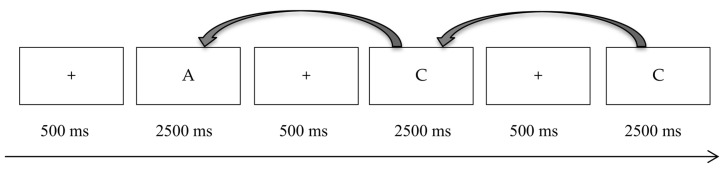
Flow chart of the 1-back task.

The 1-back task is considered an ongoing task that requires participants to compare two adjacent letters. If two letters are the same, press “J”; otherwise, press “F” (see Figure 1 for details of the 1-back task). The TBPM task is to press “1” button every minute. During the execution of ongoing tasks, participants need to press the 1 key every 1 min, which is the TBPM task. If the participant wanted to know how much time had passed, he/she could press the space bar, and there would be a time prompt in the center of the screen, telling the participant how much time had passed.

### 2.3. Design

We adopted a single-factor between-subject experimental design with three groups: baseline group, control group, and experimental group.

### 2.4. Procedure

**Figure 2:**
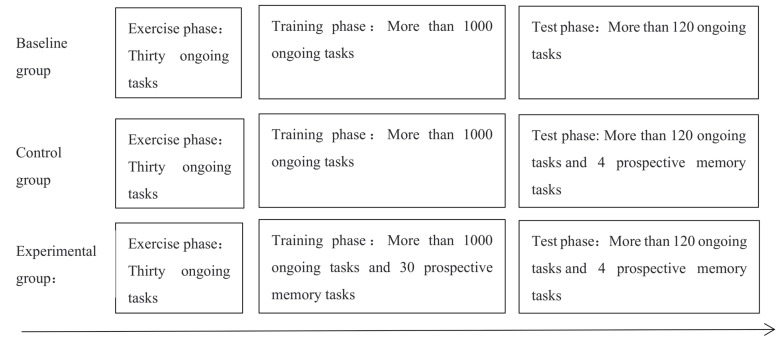
Experimental procedures of baseline group, control group and experimental group.

At the beginning of the experiment, the task requirements were explained to the participants first. This experiment program was compiled by E-Prime 2.0 software. It started with a practice phase, which presented the requirements and specific examples of the ongoing tasks, followed by 30 ongoing tasks. Each ongoing task began with a “+” fixation point in the center of the screen, which lasted for 500 ms before disappearing, and then a capital letter in the same position, which asked the participants to respond accordingly. When participants responded, the stimulus disappeared immediately. If the participants did not respond in time, the stimulus would disappear after 2500 ms and move on to the next ongoing task. The accuracy of all participants should be higher than 0.8 before the experiment could be formally started; otherwise, it needed to practice again. The formal experiment was divided into two phases: the training phase and the testing phase. The baseline group did not perform TBPM tasks all the way through and only had to perform ongoing tasks with more than 1000 trials in the training phase and more than 120 trials in the testing phase. The difference between the control group and the baseline group was the addition of four TBPM tasks during the testing phase. The difference between the experimental group and the control group was that 30 additional TBPM tasks were added during the training phase. The similarities and differences in experimental procedures between the baseline group, the control group, and the experimental group are shown in Figure 2. Participants can view the time at any time by pressing the spacebar, and how long the program has passed will be displayed on the upper center of the screen. In the testing phase, both groups were required to perform more than 120 ongoing tasks and four prospective memory tasks simultaneously.

## 3. Results

This study focused on the results after behavior training. Therefore, we only analyzed the results of the testing phase. The main indicators analyzed in this study were ongoing task performance and time monitoring frequency, which reflected the individual’s internal attention and external attention abilities, respectively, to display the individual’s attention consumption in performing TBPM tasks [17]. Time monitoring frequency reflects an individual’s dependence on external attention. The higher the time monitoring frequency, the more attention an individual pays to external time information [6]. Research on TBPM typically adopts a dual-task paradigm, where both ongoing tasks and TBPM tasks need to be executed simultaneously. The ongoing task and the TBPM task jointly occupy internal attention resources [6,17]. When TBPM tasks consume more internal attention, the performance of ongoing tasks will be correspondingly disrupted. Therefore, ongoing task performance (including accuracy and reaction speed) can indirectly reflect the internal attention consumption of TBPM.

### 3.1. TBPM Task Performance

**Figure 3:**
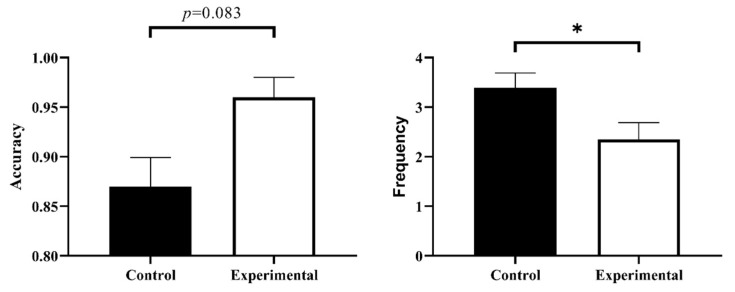
TBPM task accuracy rate (**left**) and time monitoring frequency (**right**). An asterisk indicates *p* < 0.05.

If participants pressed the “1” button within 3 s (57 s to 63 s) before and after 1 min, it was counted as correctly performing the prospective memory task. The results of the analysis of variance (ANOVA) showed that there was a trend of difference in TBPM performance between the experimental group (*M* = 0.97, *SD* = 0.11) and the control group (*M* = 0.89, *SD* = 0.18), *F*(1, 46) = 3.15, *p* = 0.083, η_p_^2^ = 0.08 (see Figure 3).

### 3.2. Time Monitoring Frequency

The results of the ANOVA showed that there was a significant difference in the time monitoring frequency between the experimental and control groups (*F*(1, 46) = 4.21, *p* < 0.05, η_p_^2^ = 0.08), and the experimental group (*M* = 2.35, *SD* = 1.65) was significantly lower than the control group (*M* = 3.39, *SD* = 1.85) (see Figure 3).

### 3.3. The Accuracy of Ongoing Task

**Figure 4:**
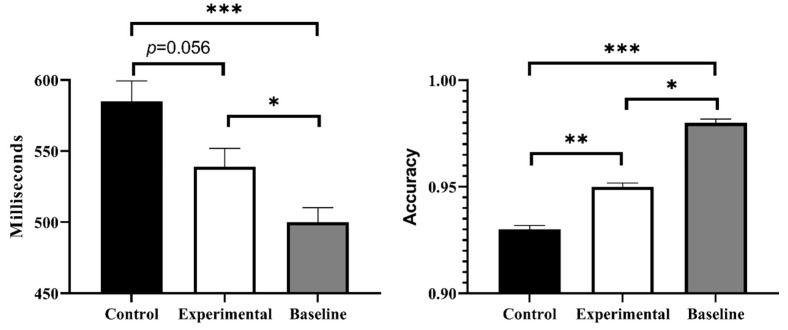
Reaction time (**left**) and accuracy rate (**right**) for ongoing task. An asterisk indicates *p* < 0.05, two asterisks indicate *p* < 0.01, and three asterisks indicate *p* < 0.001.

We found significant differences among the three groups through ANOVA: *F*(2, 70) = 14.30, *p* < 0.001, η_p_^2^ = 0.29; the baseline group (*M* = 0.98, *SD* = 0.01) performed best; the experimental group (*M* = 0.95, *SD* = 0.01) took second place; and the control group (*M* = 0.93, *SD* = 0.01) performed the worst (see Figure 4).

### 3.4. The Reaction Time of Ongoing Task

The results of the ANOVA showed significant differences in the reaction time of the ongoing task among the three groups: *F*(2, 70) = 9.31, *p* < 0.001, η_p_^2^ = 0.21. The baseline group (*M* = 500 ms, *SD* = 56 ms) had the fastest response time, which was significantly higher than the other groups. The reaction time of the experimental group (*M* = 539 ms, *SD* = 74 ms) tended to be faster than that of the control group (*M* = 585 ms, *SD* = 79 ms), *p* = 0.056. And the control group had the slowest reaction time, which was significantly higher than the baseline and experimental groups (see Figure 4).

In short, the performance of ongoing tasks in the baseline group, experimental group, and control group decreased sequentially.

## 4. Discussion

Based on daily experience, repeated practice can improve our social cognitive abilities. TBPM is a typical social cognitive ability that is very common in our daily lives. Although many studies found that repeated behavioral training could improve TBPM performance [4,5,6,7], there is currently no research focusing on how repetitive practice promotes TBPM performance. Our main purpose is to pay close attention to this issue. Based on the accuracy of the TBPM task, we found that the experimental group with repetition training tended to be better than the control group, indicating that the behavior training has a tendency to improve TBPM performance, which reflected the plasticity of TBPM and verified the results of previous studies [4,5,8].

Cognitive training tends to make individuals more “effortless” [7,22], which is also a daily experience. Existing research has found that multiple EBPM training sessions can also reduce individuals’ attention consumption and even automate EBPM task processing. So, can TBPM task training also reduce individual attention consumption like EBPM? If so, will both internal and external attention levels decrease? To what extent? This is the main issue of interest in our study. In terms of attention consumption, this study analyzed the time monitoring frequency to reflect external attention and found that the experimental group viewed the time less frequently, indicating that behavior training reduced individuals’ attention to external time information.

This study further analyzed the performance of ongoing tasks to reflect internal attention. First of all, the accuracy and reaction time of the ongoing tasks showed that, compared with the control group, the experimental group had higher accuracy and a faster response speed of ongoing tasks. After training, the individuals’ performance in ongoing tasks improved, indicating that there were more sufficient attention resources for processing ongoing tasks. At the same time, through behavioral training, TBPM tasks should reduce attention consumption, which would leave more attention resources for ongoing tasks. Therefore, behavior training reduced individuals’ attention consumption on TBPM tasks. In addition, the baseline group did not need to perform TBPM tasks throughout. Therefore, the ongoing task performance of the baseline group can be used to reflect the situation when the TBPM task is assumed to be in automatic processing. The baseline group is not affected by TBPM tasks, so the ongoing task performance of the baseline group should be the best. If the TBPM performance of the experimental group after training does not reach the level of fully automated processing, the ongoing tasks of the experimental group will be affected by TBPM. In this case, we will find that the ongoing task performance of the experimental group is worse than that of the baseline group. This study further compared the accuracy and reaction time of ongoing tasks between the experimental group and the baseline group and found that the experimental group had lower accuracy and slower reaction speed than those of the baseline group, indicating that although behavior training reduced attention consumption on TBPM tasks, it still could not promote TBPM task processing to reach an automatic level. This finding corroborates the previous research’s viewpoint, suggesting that TBPM tasks always require self-initiated attention resources [23,24]. The overall performance of the ongoing tasks indicated that behavior training also reduced the level of internal attention.

This study found that behavior training can simultaneously reduce individuals’ internal and external attention levels, which confirms our previous hypothesis and is also consistent with daily life experience. In addition, TBPM mainly includes time information processing and retrospective memory [12]. The TBPM task set in this study was to press the key “1” once every 1 min, and participants were asked to press the “1” key several times at the beginning of the experiment to familiarize themselves with the TBPM response. Such a setting minimizes the difficulty of the intended content of the TBPM task to weaken the role of retrospective memory in the TBPM task, thus highlighting the role of time information processing. Therefore, this study found that the decrease in attention consumption caused by behavior training should be mainly attributed to time information processing. So why did behavior training reduce attention consumption of time information processing? This may be related to the improvement of internal time estimation abilities. Time estimation ability is the main time information processing ability involved in TBPM. Repeated behavior training in TBPM tasks can significantly improve an individual’s time estimation ability [25], which is an ability related to internal attention. After behavior training, individuals can rely more on better time estimation abilities and reduce their dependence on external time information, thus reducing the level of external attention. At the same time, repeated training will reduce the attention expenditure of prospective memory on goal maintenance, which will leave more attention resources available for time estimation in TBPM. More attention is beneficial for improving the accuracy of time estimation [26], which promotes the execution of TBPM tasks. Furthermore, this study found that the level of internal attention also decreased, which to a certain extent suggested that the improvement of internal time information processing ability would also reduce the individuals’ dependence on attention resources. Finally, behavioral training may also promote TBPM performance through the improvement of other related abilities, such as working memory and attention allocation. Working memory is the most fundamental executive function involved in the processing of prospective memory and participates in all stages of prospective memory processing [27]. The improvement of working memory ability is equivalent to having more cognitive resources available for processing prospective memory, which is beneficial for improving prospective memory performance [28]. There is evidence to suggest that repeating a large number of prospective memory tasks can improve prospective memory performance by improving working memory ability [29]. TBPM is a prospective memory with time cues, and the improvement of working memory ability is conducive to more accurate estimation of time cues [27]. Therefore, repeated behavioral training may also rely on the improvement of working memory ability to promote TBPM performance. At the same time, TBPM cues become predictable due to their temporal information. When individuals repeatedly process these cues with regular temporal information, they can better predict when expected goals will appear, thus improving the effectiveness of temporal information processing [30,31]. Therefore, after behavioral training, individuals can form better expectations of TBPM target time points, in which case they can allocate attention more flexibly, that is, they will invest more attention closer to the target. This not only reduces attention consumption, but also improves the effectiveness of time information processing. In short, behavioral training should enhance TBPM performance through the improvement of various abilities.

This study systematically focused on the changes in attention consumption after TBPM training for the first time, which helps us better understand the mechanism of TBPM. We have three main findings. The first is that repeated behavior training can indeed improve TBPM performance, which confirms the results of previous studies [4,8]. The second is that behavior training can reduce individual attention consumption in both internal attention and external attention. The last is that behavior training cannot make TBPM task responses reach the level of automatic processing. However, there are still some shortcomings in our study. First, the intention retention interval of TBPM tasks set in this study was 1 min. Short-interval TBPLM tasks typically require continuous attention, which is easily influenced by attention. However, if the intention retention interval of TBPM tasks is long enough to be in days, months, and years, it is not realistic for individuals to continuously pay attention to time information, and the processing of time information will also be affected by the biological clock system [32,33]. Secondly, successful execution of prospective memory mainly depends on the retrieval of prospective memory cues and the extraction of intention content [34]. This study attempted to reduce the difficulty of intention content and highlight the role of time information processing through preparatory attention, while neglecting the process of extracting intention content. In real life, prospective memory tasks have longer intervals, and the intention content is also complex. Future studies should set longer delay times and increase the difficulty of ongoing tasks and intention content to improve the ecological validity of the experiment. Finally, the ongoing tasks used in this study are relatively simple and may result in ceiling effects. But time information processing is difficult to improve through short-term behavioral training under the high-difficulty condition [32]. Therefore, in the future, we can explore the effect of behavioral training on TBPM under different ongoing task difficulty conditions in order to confirm the most appropriate difficulty level for behavioral training to promote TBPM.

## 5. Conclusions

This study was the first to explore the mechanism of behavior training promoting TBPM from the perspectives of both the internal attention and the external attention. The results found that behavior training could improve TBPM performance and reduce attention consumption in both internal attention and external attention, but it could not reach the level of automatic processing. The results are consistent with our daily experience, indicating that behavioral training can enable individuals to complete social cognitive tasks well and effortlessly. The results of this study reveal the cognitive mechanism of TBPM plasticity, providing methods for promoting individual TBPM performance.

## Data Availability

The data that support the findings of this study are available from the corresponding author upon request.
